# Hypertension doctors’ awareness and practice of medication adherence in hypertensive patients: a questionnaire-based survey

**DOI:** 10.7717/peerj.16384

**Published:** 2023-11-29

**Authors:** Tao Liu, Xiexiong Zhao, Miao Huang, Yan Yang, Zhi Chen, Xin He, Xiaogang Li, Weihong Jiang

**Affiliations:** 1 Department of Cardiovascular Medicine, The Third Xiangya Hospital, Central South University, Changsha, Hunan, China; 2 Department of Cardiovascular Medicine, Xingsha hospital, Changsha, Hunan, China; 3 Department of Cardiovascular Medicine, The People’s Hospital of Liuyang, Changsha, Hunan, China; 4 Hypertension Research Center of Hunan Province, Hunan, China (Mainland)

**Keywords:** Awareness, Practice, Medication adherence, Questionnaire‑based survey, Hypertension, Hypertension doctor

## Abstract

**Background:**

Poor adherence to antihypertensive drugs is a major cause of unsatisfactory blood pressure control. Hypertension doctors play an integral role in improving medication adherence in hypertensive patients. Although most existing studies have recognized the *status quo* and influencing factors of medication adherence, little attention has been paid to hypertension doctors’ awareness and practice in hypertension management. Therefore, in this study, we aimed to investigate hypertension doctors’ awareness and practice of medication adherence in hypertensive patients.

**Methods:**

This is a cross-sectional survey. A self-reported questionnaire was developed and sent to hypertension doctors in Hunan province, China, between May 1, 2022 and July 1, 2022. Univariate and generalized linear models were used to identify the factors influencing hypertension doctors’ awareness and practice. The correlation between awareness and practice was determined using Spearman’s correlation coefficient.

**Results:**

In total, 236 valid questionnaires were collected (valid response rate, 73.5%). Of the respondents, 44.1% were chief physicians and 64.4% were females. Approximately half of the respondents were ≥40 years old and had over 14 years of working experience. Most respondents (87.7%) did not have hypertension, but 54.2% had a family history of hypertension. The average awareness and practice scores were 29.8 ± 8.8 and 39.4 ± 7.1, respectively, out of 50, with higher scores indicating higher levels of awareness or practice. More hypertension consultations and more antihypertensive prescriptions issued were associated with better awareness and practice among respondents (*ps* < 0.05). Respondents with higher education and professional titles had higher awareness (*ps* < 0.05). Moreover, respondents with 6–13 years of work experience had better practice than those with <5 years of work experience (*p* = 0.017). There was a significant correlation between hypertension doctors’ awareness and practice of medication adherence in hypertensive patients (R = 0.682, *p* < 0.001). These findings indicate that misconceptions persist in hypertension doctors’ awareness and practice of patient medication adherence.

**Conclusion:**

Hypertension doctors lack sufficient and correct awareness and practice of medication adherence in hypertensive patients.

## Introduction

Hypertension is a leading cause of cardiovascular disease and premature death worldwide (Zhou et al., 2021). Despite extensive public health education, the global prevalence of hypertension doubled from 1990 to 2019 (NCD Risk Factor Collaboration, 2021), and current control and treatment rates remain low (Zhao, 2021; Yin et al., 2022). One of the major causes of this global problem is that patients fail to take medications as advised, since most of them require long-term treatment to lower blood pressure and avoid cardiovascular diseases (Unger et al., 2020). In general, adherence to antihypertensive drugs remains the main determinant in achieving therapeutic goals (Gupta et al., 2017). Medication adherence refers to patients taking their medications as prescribed or instructed (Georges et al., 2022). There is a growing consensus on hypertension that poor medication adherence is associated with poor health outcomes in hypertensive patients, such as disease progression and increased risk of death (Menditto et al., 2020). According to reports, about 45.2% of hypertensive patients have poor medication adherence, and the rate of uncontrolled blood pressure in these patients is high (Abegaz et al., 2017; Gavrilova et al., 2019). Some related studies conducted in China found that 60.9–72.5% of participating hypertensive patients had low adherence to their medication regimens (Pan et al., 2019; Shen et al., 2020).

It has been suggested that medication adherence in patients with chronic diseases is a multidimensional and complex event, influenced by a range of factors involving the patients themselves, clinicians, public policies, and socioeconomics (Choudhry et al., 2022; Qin et al., 2020; Yeam et al., 2018; Seng et al., 2020). So far, the role of socioeconomic, patient-related, and policy-related factors has been well studied (Choudhry et al., 2022), but evaluation of clinician-related factors is still lacking. Actually, in clinical practice, hypertensive patients often consult hypertension doctors for medication adjustment. In the process of diagnosis and treatment, hypertension doctors should have a more comprehensive understanding of medication adherence than patients. Because of this, some experts suggested that hypertension doctors should find their reasons for the poor treatment effect (Rahman et al., 2015). In this sense, hypertension doctors are key enablers in improving medication adherence in hypertensive patients.

In response to the rapidly increasing burden of hypertension and related diseases, the Chinese government and public health agencies have intensified efforts to develop policies aimed at improving hypertension management (Yin et al., 2022). The Center of Hypertension Quality Control (CHQC) was established in early 2019 under the framework of the China Cardiovascular Health Alliance, with the goal of reversing the current low rate of hypertension control in China. At present, seven provincial centers have been established, among which Hunan CHQC was established in June 2019, consisted of 17 sub-centers from 10 prefecture-level cities. CHQC members come from hospitals at all levels, with large differences in age and medical experience, but they relatively represent the diagnosis and treatment level of their respective institutions in terms of hypertension control.

This study conducted a questionnaire survey among Hunan CHQC members, aiming to understand their awareness and practice of medication adherence in hypertensive patients, hoping to provide a reference for improving patients’ medication adherence.

## Materials and Methods

### Study design

The survey was completed between May 1, 2022 and July 1, 2022 in Hunan province, China. The questionnaire was filled out *via* Questionnaire Star (https://www.wjx.cn). If conceptual issues such as external interventions, patient factors, socioeconomic factors, medical factors, and policy factors were involved, a brief explanation of these concepts were provided after the question. All participants can only complete the questionnaire once. CHQC members were contacted to answer an initial online screening questionnaire. Those who met the inclusion criteria were immediately invited to participate in the main online survey.

### Inclusion criteria

All members of the Hunan CHQC were selected for this cross-sectional survey. Only those hypertension doctors who had seen hypertensive patients on long-term medication were included in this study. However, hypertension doctors engaged in studies related to medication adherence were excluded from the study. According to the evaluation standards of China’ tertiary hospitals (National Health Commission of the People’s Republic of China, Medical Administration Department, 2022), Chinese hospitals are organized into a 3-tier system to reflect their abilities in healthcare, medical education, and medical research. Distinguished by the number of beds, hospitals are designated as primary (I class, <100 beds), secondary (II class, 100–500 beds), and tertiary institutions (III class, >500 beds). Further, each tier is further subdivided into three subsidiary levels: A, B, and C based on service level, size, medical technology, medical equipment, management level, and medical quality. In this study, the respondents/CHQC members were from different levels of hospitals. As a rule of thumb, overfitting is least possible to occur if the number of samples is 10 times or more the number of independent predictors (Concato, Feinstein & Holford, 1993). Since the items used in the study consist of 10 items each for awareness-related scales and practice-related scales, the sample size for this study should be greater than 100.

### Data collection

First, to build the initial item pool, we used the keywords “hypertension/doctor/medication adherence/awareness/practice” to search five English databases (PubMed, Embase, the Cochrane Library, EBSCO, and Web of Science) and four Chinese databases (Chinese Biomedical Literature Database (CBM), China National Knowledge Infrastructure (CNKI), Wanfang Data, and VIP Information). After reading the relevant literature, a questionnaire item pool was formed, which mainly included two dimensions and 40 items.

Then, the questionnaire items were determined according to the results of expert consultation. The inclusion criteria for selecting experts were as follows: (1) Familiar with doctors’ awareness and practice of medication adherence in hypertensive patients; (2) fully understand the questionnaire preparation method and process; and (3) hold a title of deputy director or above. Finally, eight experts were selected to form an expert group, including three chief physicians, three deputy chief physicians, and two directors. They had expertise in cardiology or public administration. Basic information of these experts: three males, five females, aged 35–55 years old, and work experience ≥10 years. They all received a doctor’s degree. Two rounds of expert consultation were conducted. The first round was to screen the items of the questionnaire. The items of expert concern were selectively modified, merged, or deleted. The experts could add, remove, or modify the items. The second round was mainly based on the feedback from the results of the first round of expert consultation to modify the items and formulate the questionnaire again. In the end, 10 out of 40 items were excluded. This resulted in an initial version of the questionnaire consisting of two topics and 30 items, 24 of which were scored using the Likert scale.

Next, we calculated the correlations between individual items and total scale scores of the initial version. Items with an item-total correlation coefficient of less than 0.35 were excluded (Liu et al., 2022). After that, the scale retained 20 items, including 10 items of awareness (three questions) and 10 items of practice (four questions), with scores of 5, 4, 3, 2, and 1 based on the 5-point Likert scale. These scores were aggregated to obtain an overall awareness or practice score from 10 to 50, with higher scores reflecting higher levels of awareness or practice. The final questionnaire was divided into four parts (Table S1): demographic information, 10 items of awareness (three questions, Table S2), 10 items of practice (four questions, Table S3) and six items that were not scored on the Likert scale. All the statements are correct. The questionnaire didn’t have previously been published elsewhere.

After that, a pre-survey was conducted to further ask advice on the appropriateness of the content and format of the items so that all items are easily understandable. To this end, 100 hypertension doctors were recruited from Changsha, Hunan. They read the whole questionnaire and were asked to recount the meaning of each item. All items in the questionnaire were retained because they had appropriate statements. Cronbach’s alpha for awareness-related scales was 0.764 and Cronbach’s alpha for practice-related scales was 0.836, indicating the questionnaire has good reliability.

Finally, in our formal survey, the questionnaire had high reliability, with Cronbach’s alpha coefficients of 0.916 and 0.911 for the awareness-related and practice-related scales, respectively.

### Data analysis

Statistical analysis was performed using IBM SPSS version 24.0 (SPSS Inc., Chicago, IL, USA). Categorical variables were evaluated using frequencies and percentages; continuous variables were evaluated using mean and standard deviation. Age and work experience categories were analyzed by calculating lower and higher quartile values. The Kolmogorov–Smirnov test was performed to check data normality. Missing data was not analyzed.

Univariate analysis was performed using the chi-square test or spearman’s correlation coefficient, a nonparametric test for comparing unequal variances. Multivariate analysis was performed using a generalized linear model due to non-normally distributed data. Correlation analyses were performed to preselect the covariates to be used and to avoid those that would result in multicollinearity within the same model, and age and work experience were found to be collinear variables. In the multivariate analysis, we included the variables that were statistically significant different in the univariate analysis and all factors that may affect the dependent variables (other than age).

Spearman’s correlation coefficient were used to analyze the correlation between awareness scores and practice scores. A difference was considered statistically significant at *p* < 0.05.

### Institutional review board statement

This study was conducted in accordance with the Declaration of Helsinki and was approved by the Ethics Review Committee of the Third Xiangya Hospital of Central South University (No. I22078). Participants received an informed consent reminder prior to the start of the questionnaire, stating that continuing to answer the questionnaire implies informed consent.

## Results

### Demographic characteristics

A total of 321 hypertension doctors were contacted *via* E-mail, and 251 (78.19%) responded and completed their questionnaires. Due to incomplete information, we excluded 15 questionnaires and finally included 236 (73.5%) valid questionnaires for analysis (Table 1). A total of 64.4% of respondents were females. Approximately half of the respondents were 40 years old or above and had over 14 years of work experience. Most (87.7%) did not have hypertension, but 54.2% of them had a family history of hypertension. These respondents included 104 chief physicians.

**Table 1:** Characteristics of study participants.

Demographic characteristics	Variables	Number (%) *n* = 236
Gender	Male	84 (35.6%)
	Female	152 (64.4%)
Age, years	≤30	63 (26.7%)
	31–39	62 (26.3%)
	40–46	54 (22.9%)
	≥47	57 (24.2%)
Work experience, years	≤5	62 (26.3%)
	6–13	57 (24.2%)
	14–24	62 (26.3%)
	≥25	55 (23.3%)
Education and training	Doctor’s degree	30 (12.7%)
	Master’s degree	88 (37.3%)
	Bachelor’s degree and below	118 (50%)
Hospital level	Provincial-level Grade III-A	91 (38.6%)
	City-level Grade III-A	50 (21.2%)
	Grade III-B	38 (16.1%)
	Grade II or below	57 (24.1%)
Professional ranks	Residents	60 (25.4%)
	Attending physicians	72 (30.5%)
	Chief physicians	104 (44.1%)
History of hypertension	No	207 (87.7%)
	Yes	29 (12.3%)
Family history of hypertension	No	108 (45.8%)
	Yes	128 (54.2%)
The number of consulting for hypertension per week	>50	17 (7.2%)
	40–49	41 (17.4%)
	30–39	38 (16.1%)
	20–29	47 (19.9%)
	<20	93 (39.4%)
The number of antihypertensive prescriptions issued per week	>50	12 (5.1%)
	40–49	27 (11.4%)
	30–39	35 (14.8%)
	20–29	43 (18.2%)
	<20	119 (50.4%)

### The awareness of medication adherence

Only 49.2% of respondents completely knew the definition of medication adherence. Besides, approximately 3.8–26.7% of physicians reported that they had received relevant training on medication adherence through various means such as academic literature, online academic conferences, onsite lectures and peer discussions, hypertension research, and refresher training. Generally, these participating hypertension doctors showed insufficient understanding of medication adherence evaluation tools, with a complete comprehension rate of 5.9–8.1% (Table 2).

**Table 2:** Answers of awareness-related scale.

Question	Item	Answer, *n* (%)
Do you know the definition of medication adherence?	Completely	116 (49.2%)
	Greatly	62 (29.3%)
	Mildly	44 (18.6%)
	Slightly	10 (4.2%)
	Not at all	4 (1.7%)
What training have you received? (Excluding specialized courses such as internal medicine)		
Academic literature.	Always	63 (26.7%)
	Frequently	86 (36.4%)
	Sometimes	62 (26.3%)
	Occasionally	22 (9.3%)
	Never	3 (1.3%)
Online academic conferences.	Always	37 (15.7%)
	Frequently	59 (25.0%)
	Sometimes	75 (31.8%)
	Occasionally	54 (22.9%)
	Never	11 (4.7%)
Onsite lectures and peer discussions.	Always	45 (19.1%)
	Frequently	79 (33.5%)
	Sometimes	72 (30.5%)
	Occasionally	33 (14.0%)
	Never	7 (3.0%)
Participation of hypertension research.	Always	29 (12.3%)
	Frequently	28 (11.9%)
	Sometimes	43 (18.2%)
	Occasionally	76 (32.2%)
	Never	60 (25.4%)
Implementation of patient education	Always	32 (13.6%)
	Frequently	50 (21.2%)
	Sometimes	47 (19.9%)
	Occasionally	57 (24.2%)
	Never	50 (21.2%)
Refresher training.	Always	9 (3.8%)
	Frequently	20 (8.5%)
	Sometimes	51 (21.6%)
	Occasionally	67 (28.4%)
	Never	89 (37.7%)
What tools do you know about assessing medication adherence?		
Scales such as MMAS-8.	Completely	15 (6.4%)
	Greatly	38 (16.1%)
	Mildly	65 (27.5%)
	Slightly	65 (20.3%)
	Not at all	70 (29.7%)
Regulatory systems of medication	Completely	14 (5.9%)
	Greatly	40 (16.9%)
	Mildly	56 (23.7%)
	Slightly	66 (28.0%)
	Not at all	60 (25.4%)
Detection of biochemical indicators.	Completely	19 (8.1%)
	Greatly	37 (15.7%)
	Mildly	62 (26.3%)
	Slightly	71 (30.1%)
	Not at all	47 (19.9%)

The average awareness score of the total study subjects was 29.8 ± 8.8. Univariate analysis revealed significant differences in the awareness scores of hypertension doctors of different ages, work experience, education and training, hospital levels, professional ranks, history of hypertension, family history of hypertension, number of consulting for hypertension per week, and number of antihypertensive prescriptions issued per week (*ps* < 0.05) (Table S4). Further multivariate analysis showed that respondents with higher educational level and professional rank had higher awareness. Moreover, respondents with more consultations and antihypertensive prescriptions issued had higher awareness (*ps* < 0.05) (Table 3).

**Table 3:** Factors associated with awareness. Bolded *p*-values indicate statistical significance.

Variable		β	95% CI	*p*
Work experience	≤5	Ref		
	6–13	−0.703	[−2.956 to 1.551]	0.541
	14–24	−1.611	[−4.236 to 1.014]	0.229
	≥25	−2.510	[−5.419 to 0.400]	0.091
Education and training	Doctor’s degree	Ref		
	Master’s degree	−2.435	[−4.399 to −0.470]	**0.015**
	Bachelor’s degree and below	−2.558	[−4.669 to −0.448]	**0.017**
Hospital level	Provincial-level Grade III-A	Ref		
	City-level Grade III-A	1.385	[−0.260 to 3.029]	0.099
	Grade III-B	−0.016	[−1.822 to 1.790]	0.986
	Grade II or below	−0.703	[−2.384 to 0.978]	0.536
Professional ranks	Residents	Ref		
	Attending physicians	1.924	[−0.310 to 4.157]	0.091
	Chief physicians	3.279	[0.537–6.020]	**0.019**
History of hypertension	No	Ref		
	Yes	−0.192	[−1.943 to 1.559]	0.830
Family history of hypertension	No	Ref		
	Yes	0.472	[−0.640 to 1.585]	0.405
The number of consulting for hypertension per week	<20	Ref		
	20–29	4.876	[3.171 to 6.582]	**<0.001**
	30–39	5.517	[2.887 to 8.148]	**<0.001**
	40–49	8.690	[5.362 to 12.018]	**<0.001**
	≥50	11.209	[6.920 to 15.498]	**<0.001**
The number of antihypertensive prescriptions issued per week	<20	Ref		
	20–29	3.658	[1.440 to 5.875]	**<0.001**
	30–39	4.930	[1.995 to 7.865]	**<0.001**
	40–49	7.033	[3.505 to 10.561]	**<0.001**
	≥50	13.717	[9.103 to 18.330]	**<0.001**

Regarding the percentage of patient medication adherence, 41.1% of respondents selected approximately half of patients had good medication adherence and only 5.5% of respondents selected >80% patients had good medication adherence. When asked to explain poor medication adherence in detail, 75.4% of respondents selected all of the conditions listed, including not taking medication on time, unauthorized dosage changes, and unauthorized changes in dosing frequency. Regarding the understanding of the factors affecting medication adherence, only 41.1% of respondents answered that medical factors were very important, while most (75.1%) considered patient factors to be very important. In addition, 31.4% and 29.2% of respondents rated socioeconomic and policy factors, respectively, as very important. When asked which clinicians were primarily responsible for patient medication adherence, specialists, general practitioners, and nurses accounted for 21.2%, 67.4%, and 3.4%, respectively. Nevertheless, 72.5% of respondents agreed that medication adherence can be improved through external interventions (Table 4).

**Table 4:** Answers of supplementary questions.

Question	Item	Answers of yes, *n* (%)
Which of the following situations do you think can be considered as poor medication adherence of hypertension patients?	Not taking medications on time	231 (97.9%)
	Unauthorized dosage changes	221 (93.6%)
	Unauthorized changes in dosing frequency	224 (94.9%)
	Self withdrawal medications	229 (97.0%)
	Omission of medications	208 (88.1%)
	Not buying medications in time	196 (93.1%)
	All the above situations	178 (75.4%)
Please rank the following factors that affect the patient’s medication adherence according to the importance you think.	Patient factors	Very important, 180 (76.3%)
		Important, 41 (17.4%)
		Moderate important, 12 (5.1%)
		Somewhat important, 3 (1.3%)
		Not important, 0
	Medical factors	Very important, 97 (41.1%)
		Important, 101 (42.8%)
		Moderate important, 31 (13.1%)
		Somewhat important, 7 (3.0%)
		Not important, 0
	Socioeconomic factors	Very important, 74 (31.4%)
		Important, 102 (43.2%)
		Moderate important, 52 (22.0%)
		Somewhat important, 8 (3.4%)
		Not important, 0
	Policy factors	Very important, 69 (29.2%)
		Important, 88 (37.3%)
		Moderate important, 63 (26.7%)
		Somewhat important, 15 (6.4%)
		Not important, 1 (0.4%)
Who do you think should be the main responsible for hypertensive patient’s medication adherence?	Specialists	50 (21.2%)
	General practitioners	159 (67.4%)
	Nurses	8 (3.4%)
	Others	19 (8.1%)
Do you think medication adherence can be improved by external intervention?	Yes	171 (72.5%)
	No	65 (27.5%)
What percentage of hypertensive patients do you think have good medication adherence?	<20%	21 (8.9%)
	20–39%	52 (22.0%)
	40–59%	97 (41.1%)
	60–80%	53 (22.5%)
	>80%	13 (5.5%)

### The practice of medication adherence

Nearly 60.0% of respondents said they had been emphasizing the importance of medication adherence, while approximately 25% of them had been evaluating the medication adherence of hypertensive patients. The interventions always used by respondents to improve medication adherence include: “Answering the patients’ questions” (35.2%), “Emphasis on the role of medication” (52.1%), “Requirements for regular outpatient follow-up” (51.7%), and “Requirements for accompanying/family members to observe the patient’s medication” (41.1%). Only a small portion of respondents always used apps (20.3%), video, and audio materials (14.8%) to educate hypertensive patients. Furthermore, 33.5% of respondents always took the regimen of minimizing the number of doses. In addition, 78.8% of the respondents always or frequently took the individual differences of patients into account when intervening in medication adherence (Table 5).

**Table 5:** Answers of practice-related scale.

Question	Item	Answer, *n* (%)
How often did you evaluate medication adherence?	Always	59 (25.0%)
	Frequently	68 (28.8%)
	Sometimes	71 (30.1%)
	Occasionally	27 (11.4%)
	Never	11 (4.7%)
How often did you highlight the importance of medication adherence for hypertensive patients?	Always	139 (58.9%)
	Frequently	68 (28.8%)
	Sometimes	22 (9.3%)
	Occasionally	5 (2.1%)
	Never	2 (0.8%)
What interventions to improve medication adherence have you used?		
Apps that facilitate patients’ self-management of blood pressure	Always	48 (20.3%)
	Frequently	42 (17.8%)
	Sometimes	61 (25.8%)
	Occasionally	51 (21.6%)
	Never	34 (14.4%)
Audio and video materials for missions.	Always	35 (14.8%)
	Frequently	67 (28.4%)
	Sometimes	70 (29.7%)
	Occasionally	42 (17.8%)
	Never	22 (9.3%)
Answering the patients’ questions.	Always	83 (35.2%)
	Frequently	92 (39.0%)
	Sometimes	42 (17.8%)
	Occasionally	16 (6.8%)
	Never	3 (1.3%)
Emphasis on the role of medication	Always	123 (52.1%)
	Frequently	91 (38.6%)
	Sometimes	17 (7.2%)
	Occasionally	2 (0.8%)
	Never	3 (1.3%)
Regimens that minimize the number of doses.	Always	79 (33.5%)
	Frequently	97 (41.1%)
	Sometimes	44 (18.6%)
	Occasionally	11 (4.7%)
	Never	5 (2.1%)
Requirements for regular outpatient follow-up.	Always	122 (51.7%)
	Frequently	97 (41.4%)
	Sometimes	10 (4.2%)
	Occasionally	5 (2.1%)
	Never	2 (0.8%)
Requirements for escorts/families to observe the patient’s medication.	Always	97 (41.1%)
	Frequently	96 (40.7%)
	Sometimes	33 (14.0%)
	Occasionally	8 (3.4%)
	Never	2 (0.8%)
How often did you take individual differences into account when implementing interventions to improve medication adherence?	Always	98 (41.5%)
	Frequently	88 (37.3%)
	Sometimes	42 (17.8%)
	Occasionally	6 (2.5%)
	Never	2 (0.8%)

The average practice score of the respondents was 39.4 ± 7.1. Univariate analysis found significant differences in the practice scores of hypertension doctors with different ages, work experience, education and training, hospital levels, professional ranks, history of hypertension, family history of hypertension, number of consulting for hypertension per week, and number of antihypertensive prescriptions issued per week (*ps* < 0.05) (Table S5). Multivariate analysis showed that respondents with more consultations for hypertension and antihypertensive prescriptions issued had better practice (*ps* < 0.05). Also, respondents with 6–13 years of work experience had better practice than those with less than 5 years of work experience (*p* = 0.017) (Table 6).

**Table 6:** Factors associated with practice. Bolded *p*-values indicate statistical significance.

Variable		β	95% CI	*p*
Work experience	≤5	Ref		
	6–13	2.941	[0.531–5.351]	**0.017**
	14–24	2.599	[−0.209 to 5.406]	0.070
	≥25	2.200	[−0.912 to 5.312]	0.166
Education and training	Doctor’s degree	Ref		
	Master’s degree	0.249	[−1.851 to 2.350]	0.816
	Bachelor’s degree and below	1.368	[−0.888 to 3.625]	0.235
Hospital level	Provincial-level Grade III-A	Ref		
	City-level Grade III-A	0.243	[−1.515 to 2.002]	0.786
	Grade III-B	−0.233	[−2.164 to 1.699]	0.813
	Grade II or below	−1.148	[−2.946 to 0.650]	0.211
Professional ranks	Residents	Ref		
	Attending physicians	0.809	[−1.579 to 3.198]	0.507
	Chief physicians	−0.461	[−3.393 to 2.470]	0.758
History of hypertension	No	Ref		
	Yes	1.124	[−0.749 to 2.996]	0.240
Family history of hypertension	No	Ref		
	Yes	1.136	[−0.054 to 2.326]	0.061
The number of consulting for hypertension per week	<20	Ref		
	20–29	3.924	[2.100–5.748]	**<0.001**
	30–39	6.580	[3.767–9.393]	**<0.001**
	40–49	8.891	[5.331–12.450]	**<0.001**
	≥50	10.355	[5.768–14.942]	**<0.001**
The number of antihypertensive prescriptions issued per week	<20	Ref		
	20–29	1.475	[−0.897 to 3.846]	0.223
	30–39	1.035	[−2.104 to 4.174]	0.518
	40–49	3.806	[0.033–7.579]	**0.048**
	≥50	5.089	[0.155–10.023]	**0.043**

**Figure 1:**
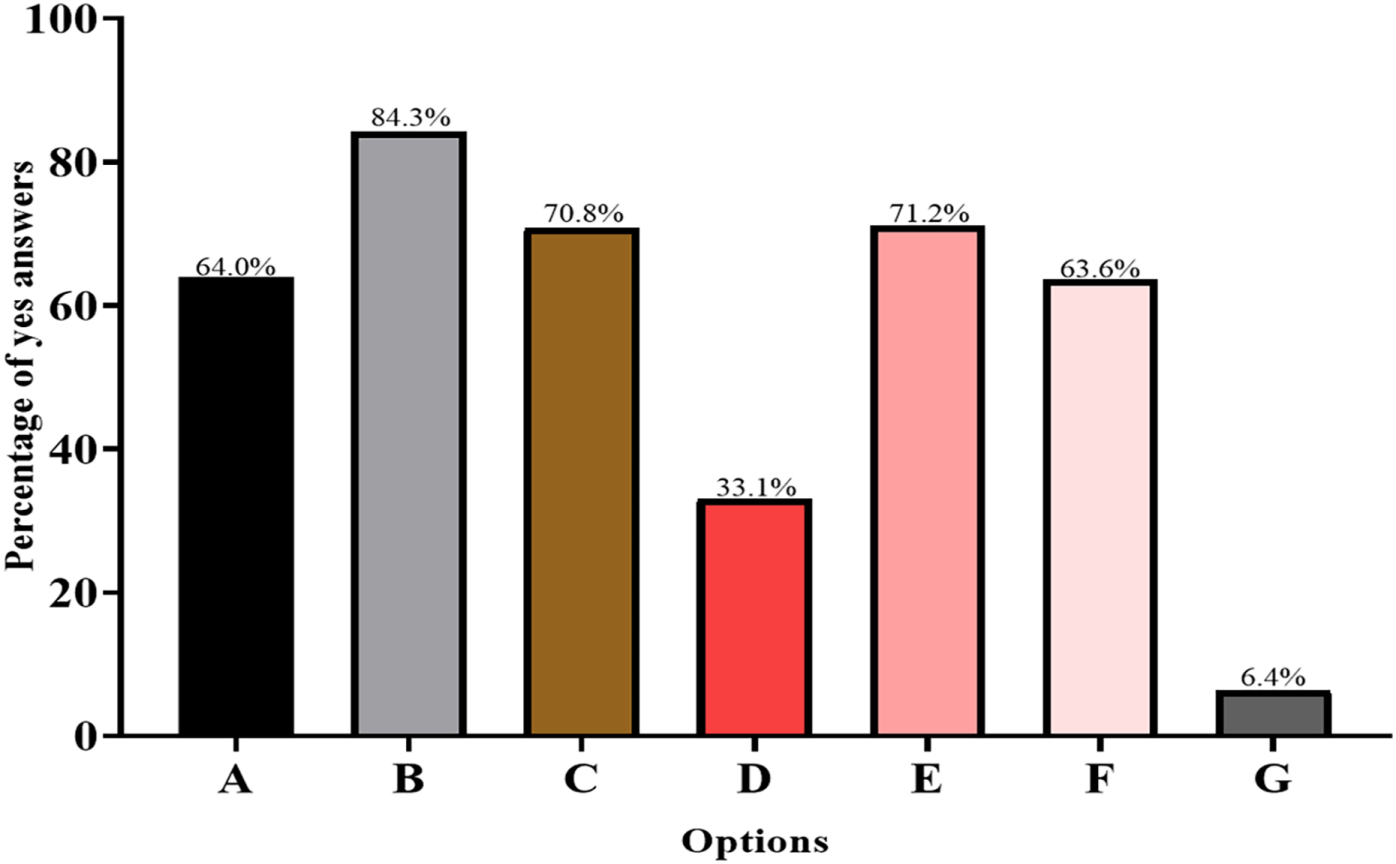
Obstacles in improving medication adherence. (A) Lack of cooperation. (B) Heavy clinical work. (C) Visits not for hypertension. (D) Strained doctor-patient relation. (E) Poor doctor-patient communication. (F) Lack of knowledge. (G) Others. The y-axis represents percentage of choices, and x-axis represents questionnaire items.

Next, we investigated potential obstacles to improving medication adherence in hypertensive patients. The results showed that “heavy clinical work” (84.3%) and “poor doctor-patient communication” (71.2%) were the two main obstacles. Additionally, 64.0% of the respondents indicated that the lack of cooperation between hypertension doctors and patients was the main resistance to improving medication adherence (Fig. 1).

### Relevance of medication adherence awareness and practice

**Figure 2:**
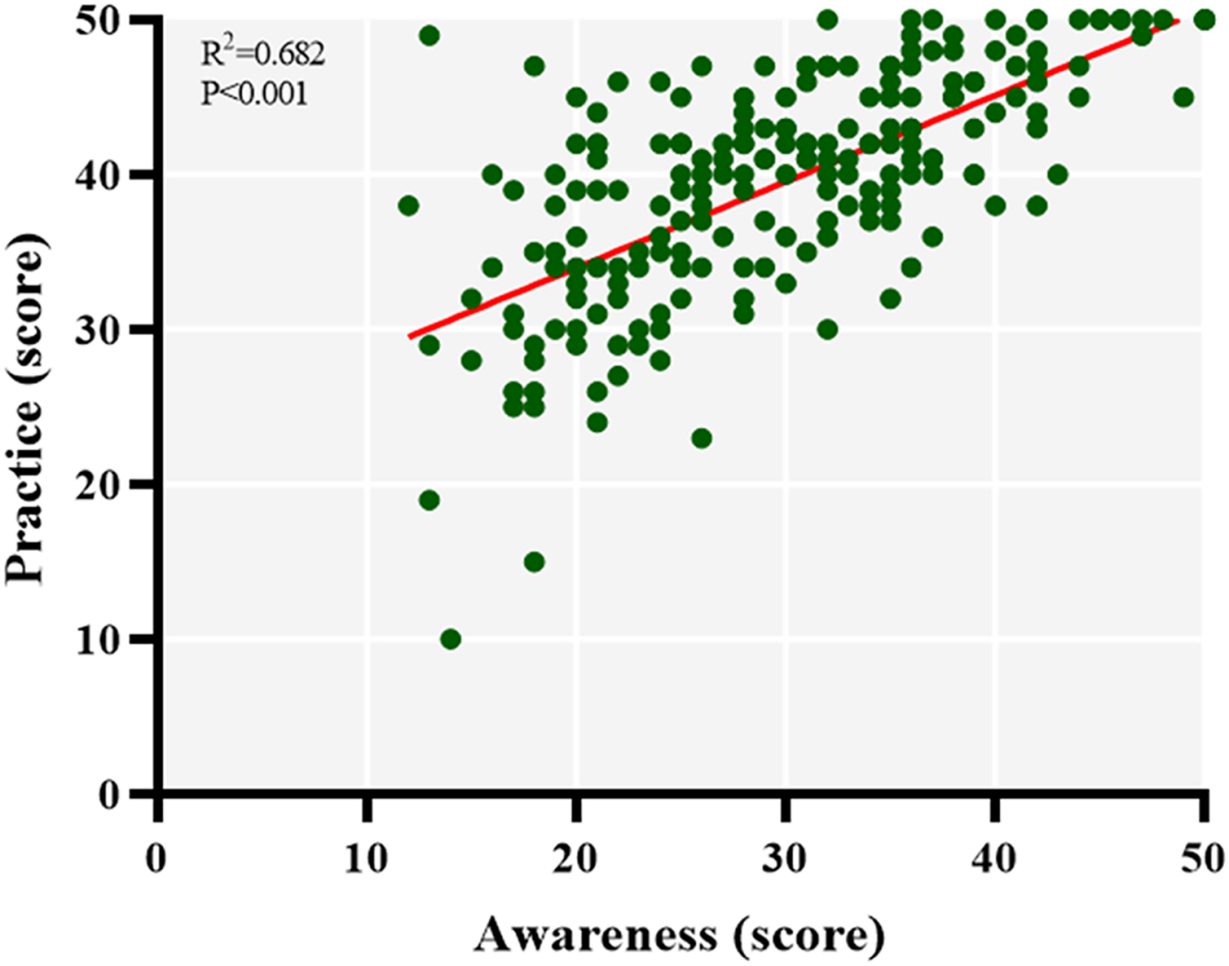
Relevance of awareness and practice of medication adherence. The y-axis represents the situation of practice, and x-axis represents the situation of awareness.

Finally, we analyzed the correlation between awareness scores and practice scores using the Spearman correlation coefficient and found a strong positive correlation between hypertension doctors’ awareness and practice in terms of patient medication adherence (R = 0.682, *p* < 0.001), suggesting that the higher their awareness, the better their practice (Fig. 2).

## Discussion

We found that hypertension doctors’ awareness and practice of medication adherence in their hypertensive patients were far from ideal, which is related to the number of consultations for hypertension, the number of antihypertensive prescriptions issued, educational level, and professional rank. Generally, hypertension doctors who had more outpatient visits and wrote more antihypertensive prescriptions had better awareness and practice (*ps* < 0.05). Moreover, senior and highly educated hypertension doctors had higher awareness, while junior and less educated hypertension doctors had lower awareness (*ps* < 0.05). Also, we found that a considerable degree of misconceptions still existed in hypertension doctors regarding the awareness and practice of medication adherence in hypertensive patients.

To our knowledge, this is the first survey of Chinese hypertension doctors’ awareness and practice of medication adherence in hypertensive patients. This self-developed questionnaire will be of great help to assess and quantify hypertension doctors’ awareness and practice of patient medication adherence. Although a related report has been published (Barbouni et al., 2017), a major difference between the previous report and our study is that the former focused on compliance-promoting interventions at the patient level. Not only did we use a self-made scale to measure hypertension doctors’ awareness and practice of patient medication adherence, but we also recruited subjects working at different levels of hospitals, mainly including those with long-term experience in managing hypertension.

Our survey results showed that the average awareness scores and practice scores were 29.8 ± 8.8 and 39.4 ± 7.1 out of 50, respectively, suggesting that there are major deficiencies in the current management of hypertension. First, most respondents (50.8%) knew little about the definition of medication adherence. It may be related to the fact that most hypertension doctors are committed to the improvement of professional quality rather than medical humanities and related knowledge, and there are few medical humanities education courses offered by medical colleges in mainland China (Qian et al., 2018). On the other hand, the premise of fully understanding medication adherence is to evaluate it correctly and objectively, but we found that most of the respondents were not familiar with objective assessment tools such as the 8-item Morisky Medication Adherence Scale (Morisky, Green & Levine, 1986), which may lead to overestimation of patients’ medication adherence. In this study, we found that 69.1% of hypertension doctors believed that their hypertensive patients had a good medication adherence of >40%, which is higher than previous findings in China (Shi et al., 2019; Gao et al., 2020; Ding et al., 2018). The regularity of practice appears to follow the same trends with awareness. Hypertension doctors who consulted more hypertensive patients and prescribed more antihypertensive drugs had better practice.

A total of 41.1% of respondents answered that medical factors were very important among the various factors affecting medication adherence of hypertensive patients. These hypertension doctors are not only the makers of medication regimens, but also responsible for supervising the rational drug use. In this sense, hypertension doctors have better room for improvement than patients. As for which level of hospital physicians are mainly responsible for the medication adherence of hypertensive patients, 67.4% of the respondents believed that they were general practitioners. Considering the different medical services and responsibilities of specialist cardiologists and general practitioners in the health care system (Wangler & Jansky, 2023), and the strong support of national policies for primary health care facilities, general practitioners are indeed suitable to assume the main responsibility for medication adherence. For general practitioners, it has been suggested to increase relevant continuing medical education opportunities, simplify promotion channels, and improve their level of diagnosis and treatment of hypertension (Ye et al., 2020).

In this survey, hypertension doctors seldom use apps, video, and audio materials to educate hypertensive patients. However, apps that facilitate self-management of hypertension can significantly improve patient medication adherence by assisting doctors to manage health concerns and data remotely and by providing patients with personalized self-care advice (Liu, Xie & Or, 2020). Further, using apps can also create a social network among hypertensive patients, allowing them to exchange information, improve mood, and reduce stress (Mugabirwe et al., 2021). A meta-analysis reported that video-assisted patient education materials improve cardiovascular disease prevention behaviors and that use of them as a support tool for hypertension management significantly improves patient follow-up (Miller et al., 2021).

The use of single-pill combinations (SPCs) to initiate treatment can enhance medication adherence and speed up the process of reaching the blood pressure target range in hypertensive patients (Ott & Schmieder, 2022). However, most hypertension doctors in the study were unaware of the importance of SPCs for medication adherence. The importance of SPCs for patient medication adherence has been emphasized in hypertension management guidelines, including the 2017 Hypertension Guidelines published by the American College of Cardiology/American Heart Association (ACC/AHA) (Lee et al., 2018), the 2018 European Society of Cardiology/European Society of Hypertension Guideline (Whelton et al., 2022), and the International Hypertension Guidelines published by International Society of Hypertension (Nugroho et al., 2022), all of which recommend SPCs as the optimal choice for initial antihypertensive treatment.

This study showed that “heavy clinical work” (84.3%) and “poor doctor-patient communication” (71.2%) were the two main obstacles to improving the medication adherence of hypertensive patients, which is consistent with the current status of diagnosis and treatment in China (Zhang et al., 2021). A review of the average physician consultation time found that the latest reported mean consultation length of Chinese physicians was 5 min or less, ranked third last out of 67 countries (Irving et al., 2017). The reason for such short physician consultation time may reflect several factors, including issues relating to governance, workforce, access, continuity, comprehensiveness, and coordination (Irving et al., 2017). Moreover, since there is no appointment system in China, physicians may consult over 90 times a day, involving multiple symptoms or diseases, so it takes a considerable amount of time to provide repeat prescriptions (Jin et al., 2015). Such heavy clinical work squeezes the self-study time of physicians, making them unable to focus on additional services beyond the diagnosis and treatment of hypertensive patients. Medication adherence can be affected at different phases of hypertension control (Choudhry et al., 2014). Regardless, effective physician-patient communication is important, both at the beginning of a patient visit and during follow-up.

There are some potential limitations to extending our findings to broader health system settings. First, most of the survey’s participants were from the provincial health care systems, factors that influence the generalizability to other regional and different types of healthcare systems must be considered. However, in China, many aspects of health service integration, including informational continuity, have been shown to be consistent across different system types, thereby reducing this concern. The second limitation is the potential for respondents to misinterpret survey questions. Likert scale questions are based on perception and their interpretation is complex (Asfuroğlu et al., 2022). Although some respondents may interpret these questions differently than others, the problem of interpreting survey questions is an unavoidable aspect of survey research. However, respondents to our pre-survey reported no difficulty understanding or answering these questions. Lastly, hypertension doctors were free to participate and complete the survey. Thus, it cannot be ruled out that this self-selected sample was subject to selection bias, with participants being more interested in medication adherence.

Despite the above limitations, this study, as the first survey of Chinese hypertension doctors on the awareness and practice of medication adherence in hypertensive patients, still has certain enlightening significance. This will prompt us to conduct a more in-depth study of the relevant conclusions drawn from this study.

## Conclusions

The survey results show that the average awareness score and practice score was 29.79 ± 8.78 and 39.42 ± 7.13 out of 50, respectively, suggesting that there is still a lot of room for improvement. We found that hypertension doctors’ lack of sufficient and correct awareness and practice of hypertensive patients’ medication adherence was related to the number of hypertension consultations, the number of antihypertensive prescriptions issued, educational level, and professional rank. Reducing clinical workload and correspondingly strengthening the training of doctor-patient communication skills may help improve the medication adherence of hypertensive patients. Equally important, continuing medical education should include courses that improve hypertension doctors’ awareness and practice of medication adherence in hypertensive patients.

## Supplemental Information

10.7717/peerj.16384/supp-1 Supplemental Information 1 Raw data.

10.7717/peerj.16384/supp-2 Supplemental Information 2 Questionnaire (Chinese version).

10.7717/peerj.16384/supp-3 Supplemental Information 3 Questionnaire.

10.7717/peerj.16384/supp-4 Supplemental Information 4 Likert-scale questions of awareness.

10.7717/peerj.16384/supp-5 Supplemental Information 5 Likert-scale questions of practice.

10.7717/peerj.16384/supp-6 Supplemental Information 6 Univariate analysis of awareness.

10.7717/peerj.16384/supp-7 Supplemental Information 7 Univariate analysis of practice.

10.7717/peerj.16384/supp-8 Supplemental Information 8 The research title, methods, study population, results, and conclusion.

## Additional Information and Declarations

### Data Availability

The following information was supplied regarding data availability: The raw measurements are available in the Supplemental Files.
