# Supplementary material for: Hypertension doctors’ awareness and practice of medication adherence in hypertensive patients: a questionnaire-based survey
Source: PeerJ. 2023 Nov 29;11:e16384. doi: 10.7717/peerj.16384 (PMC10693237; doi:10.7717/peerj.16384)
Supplement: Supplemental Information 2 [file peerj-11-16384-s002.docx]

**问卷调查表**

1.您的性别是？ [单选题]

| ○男 |
| --- |
| ○女 |

2.您的年龄是？ [填空题]

_________________________________

3.您的工作年限是？ [填空题]

_________________________________

4.您的最高学历是？ [单选题]

| ○博士 |
| --- |
| ○硕士 |
| ○本科及以下  5.您的工作单位是？ [填空题]  _________________________________ |

6.您的职称等级是？ [单选题]

|  |
| --- |
| ○初级 |
| ○中级 |
| ○高级 |

7.您本人是否有高血压？ [单选题]

| ○是 |
| --- |
| ○否 |

8.您家中是否有高血压相关家族史？ [单选题]

| ○是 |
| --- |
| ○否 |

9.您每周接诊的高血压的患者数目是？ [单选题]

○＞50

○40-49

○30-39

○20-29

○＜20

10.您每周开出的高血压处方数是？ [单选题]

○＞50

○40-49

○30-39

○20-29

○＜20

11.您是否清楚药物依从性的含义？ [单选题]

| ○非常清楚 | ○清楚 | ○部分清楚 |  | ○清楚一点 | ○完全不清楚 |
| --- | --- | --- | --- | --- | --- |

12.关于如何管理高血压患者，您受过哪些相关培训？（除外《内科学》等专业课程）[矩阵量表题]

|  | 总是 | 经常 | 偶尔 | 很少 | 从不 |
| --- | --- | --- | --- | --- | --- |
| 自行阅读科普文章、专业论文等 | ○ | ○ | ○ | ○ | ○ |
| 参与线下专家授课、同行讨论等 | ○ | ○ | ○ | ○ | ○ |
| 报名线上相关讲座、学术会议等 | ○ | ○ | ○ | ○ | ○ |
| 主持或参与高血压相关研究 | ○ | ○ | ○ | ○ | ○ |
| 举办或参加患教会、病友交流会等 | ○ | ○ | ○ | ○ | ○ |
| 申请行高血压专科进修 | ○ | ○ | ○ | ○ | ○ |

13.您知道下列哪些评价药物依从性的量表/工具？[矩阵量表题]

|  | 非常清楚 | 清楚 | 部分清楚 | 清楚一点 | 完全不清楚 |
| --- | --- | --- | --- | --- | --- |
| 使用MMS-8或其他量表/问卷 | ○ | ○ | ○ | ○ | ○ |
| 计算药物持有率、使用服药监测系统 | ○ | ○ | ○ | ○ | ○ |
| 检测血或尿中药物浓度等生物化学指标 | ○ | ○ | ○ | ○ | ○ |

14.您认为下列哪些情况可以视为高血压患者药物依从性不佳？ [多选题]

| □不按时服药 |
| --- |
| □擅自增减剂量 |
| □擅自改变服药频率 |
| □自主停药 |
| □漏服药物 |
| □未及时买药  15.请为下列影响患者药物依从性的因素按您认为的重要程度进行选择[矩阵量表题]  患者因素主要包括患者文化素养不高、重视程度不够、记忆力差、不相信医生、担心不良反应、更愿意用中药、对疾病了解程度不够等； 社会经济因素主要包括费用问题及媒体的影响等； 医疗团队因素主要包括医生对患者的解释和教育不足、随访频率不足、就诊时间限制等； 体系政策的因素主要包括社区医疗卫生保健系统不健全、医保政策限制等   \|  \| 非常重要 \| 重要 \| 比较重要 \| 不太重要 \| 不重要 \| \| --- \| --- \| --- \| --- \| --- \| --- \| \| 患者自身因素 \| ○ \| ○ \| ○ \| ○ \| ○ \| \| 医疗团队因素 \| ○ \| ○ \| ○ \| ○ \| ○ \| \| 社会经济因素 \| ○ \| ○ \| ○ \| ○ \| ○ \| \| 体系政策因素 \| ○ \| ○ \| ○ \| ○ \| ○ \| |

16.您认为提高患者药物依从性所依赖的“主力军”应该是？(单选) [单选题]

| ○大型医院专科医生 |
| --- |
| ○社区医生 |
| ○护理人员 |
| ○其他人员 _________________ * |

17.您在诊疗时是否对患者药物依从性水平进行过评价？ [单选题]

| ○总是 | ○经常 | ○有时 | ○很少 | ○从不 |
| --- | --- | --- | --- | --- |

18.您在临床诊疗中是否有意识地跟患者强调药物依从性的重要性？ [单选题]

| ○总是 | ○经常 | ○有时 | ○很少 | ○从不 |
| --- | --- | --- | --- | --- |

19.您在临床中使用过哪些方式提高患者药物依从性[矩阵量表题]

|  | 总是 | 经常 | 有时 | 很少 | 从不 |
| --- | --- | --- | --- | --- | --- |
| 借助便于患者自我管理血压的App | ○ | ○ | ○ | ○ | ○ |
| 使用视频/音频材料对患者进行宣教 | ○ | ○ | ○ | ○ | ○ |
| 回答患者关于高血压相关知识包括发病机制、危害、用药方案、药物潜在副作用等 | ○ | ○ | ○ | ○ | ○ |
| 强调药物治疗的重要性 | ○ | ○ | ○ | ○ | ○ |
| 尽可能使用采取单药（或单片复方制剂）等服药次数较少的治疗方案 | ○ | ○ | ○ | ○ | ○ |
| 要求患者定期门诊随诊 | ○ | ○ | ○ | ○ | ○ |
| 要求陪护/家人观察患者服药情况 | ○ | ○ | ○ | ○ | ○ |

20.您在对患者实施提高药物依从性的干预措施时是否考虑到患者的个体化差异？ [单选题]

| ○总是 | ○经常 | ○有时 | ○很少 | ○从不 |
| --- | --- | --- | --- | --- |

21.关于高血压患者的药物依从性，您认同以下哪个观点？ [单选题]

| ○药物依从性的高低取决于患者的自觉性（外力干预基本无效）） |
| --- |
| ○药物依从性可以通过外力干预被提高 |

22.您所接触的高血压患者中，大约有多少比例可以被认为药物依从性好？（单选） [单选题]

| ○＜20% |
| --- |
| ○20%-39% |
| ○40%-59% |
| ○60%-80% |
| ○＞80% |

23.您认为下列哪些因素是医生提高患者药物依从性的阻力之一 [多选题]

| □医生对药物依从性认识不足 |
| --- |
| □缺乏其他配合者（如药师） |
| □病人数量多，临床工作繁重 |
| □高血压非本次患者就诊主要原因 |
| □医患关系紧张 |
| □医患沟通不佳 |
| □其他 _________________* |
