# Supplementary material for: Hypertension doctors’ awareness and practice of medication adherence in hypertensive patients: a questionnaire-based survey
Source: PeerJ. 2023 Nov 29;11:e16384. doi: 10.7717/peerj.16384 (PMC10693237; doi:10.7717/peerj.16384)
Supplement: Supplemental Information 3 [file peerj-11-16384-s003.docx]

Table S1 Questionnaire

1. What is your gender? [Single choice question]

○ Male

○ Female

2. What is your age? [Fill in the blank]

_________________________________

3. How long have you worked? [Fill in the blank]

_________________________________

○Doctor's degree

○Master's degree

○Bachelor's degree and below

4.What is your highest academic degree? [Single choice question]

5. What is your work unit? [Fill in the blank]

_________________________________

○Residents

○Attending physicians

○Chief physicians

6. What is your professional rank? [Single choice question]

7. Do you have history of hypertension? [Single choice question]

○ Yes

○ No

8. Do you have family history of hypertension? [Single choice question]

○ Yes

○ No

○＞50

○40-49

○30-39

○20-29

○＜20

9. What is the number of your consultations for hypertension per week? [Single choice question]

10. What is the number of your antihypertensive prescriptions issued per week? [Single choice question]

○＞50

○40-49

○30-39

○20-29

○＜20

11. Do you know the definition of medication adherence? [Single choice question]

○Completely

○Greatly

○Mildly

○Slightly

○Not at all

12. What training have you received?(Excluding specialized courses such as Internal Medicine) [Likert-scale questions]

| Academic literature. | Always  Frequently  Sometimes  Occasionally  Never |
| --- | --- |
| Online academic conferences. | Always  Frequently  Sometimes  Occasionally  Never |
| Onsite lectures and peer discussions. | Always  Frequently  Sometimes  Occasionally  Never |
| Participation of hypertension research. | Always  Frequently  Sometimes  Occasionally  Never |
| Implementation of patient education. | Always  Frequently  Sometimes  Occasionally  Never |
| Refresher training. | Always  Frequently  Sometimes  Occasionally  Never |

13. What tools do you know about assessing medication adherence？Likert-scale questions]

| Scales such as MMAS-8. | Completely  Greatly  Mildly  Slightly  Not at all |
| --- | --- |
| Regulatory systems of medication | Completely  Greatly  Mildly  Slightly  Not at all |
| Detection of biochemical indicators. | Completely  Greatly  Mildly  Slightly  Not at all |

14. Which of the following situations do you think can be considered as poor medication adherence of hypertensive patients? [Multiple choice questions]

○ Not taking medications on time

○ Unauthorized dosage changes

○ Unauthorized changes in dosing frequency

○ Self withdrawal medications

○ Omission of medications

○ Not buying medications in time

15. Please rank the following factors that affect the hypertensive patient's medication adherence according to the importance you think. [matrix scale question]

| Patient factors | Very important  Important  Moderate important  Somewhat important  Not important |
| --- | --- |
| Medical factors | Very important  Important  Moderate important  Somewhat important  Not important |
| Socioeconomic factors | Very important  Important  Moderate important  Somewhat important  Not important |
| Policy factors | Very important  Important  Moderate important  Somewhat important  Not important |

16. which clinicians were primarily responsible for patient medication adherence? [Single choice question]

○ Specialists

○ General practitioners

○ Nurses

○ Others

17. How often did you evaluate medication adherence? [Likert-scale questions]

○ Always

○ Frequently

○ Sometimes

○ Occasionally

○ Never

18. How often did you highlight the importance of medication adherence for hypertensive patients? [Likert-scale questions]

○ Always

○ Frequently

○ Sometimes

○ Occasionally

○ Never

19.What interventions to improve medication adherence have you used?[Likert-scale questions]

| Apps that facilitate patients’ self-management of blood pressure. | Always  Frequently  Sometimes  Occasionally  Never |
| --- | --- |
| Audio and video materials for missions. | Always  Frequently  Sometimes  Occasionally  Never |
| Answering the patients’ questions. | Always  Frequently  Sometimes  Occasionally  Never |
| Emphasis on the role of medication | Always  Frequently  Sometimes  Occasionally  Never |
| Regimens that minimize the number of doses. | Always  Frequently  Sometimes  Occasionally  Never |
| Requirements for regular outpatient follow-up. | Always  Frequently  Sometimes  Occasionally  Never |
| Requirements for escorts/families to observe the patient's medication. | Always  Frequently  Sometimes  Occasionally  Never |

20. How often did you take individual differences into account when implementing interventions to improve medication adherence? [Single choice question]

○ Always

○ Frequently

○ Sometimes

○ Occasionally

○ Never

21. Do you think medication adherence can be improved by external intervention？[Single choice question]

○ Yes

○ No

22. What percentage of hypertensive patients do you think have good medication adherence? [Single choice question]

○ ＜20%

○ 20%-39%

○ 40%-59%

○ 60%-80%

○ ＞80%

23. What do you think are obstacles in improving medication adherence?[Multiple choice questions]

○ Lack of cooperation

○ Heavy clinical work

○ Visits not for hypertension

○ Strained doctor-patient relation

○ Poor doctor-patient communication

○ Lack of knowledge

○ Others
