# Supplementary material for: Hypertension doctors’ awareness and practice of medication adherence in hypertensive patients: a questionnaire-based survey
Source: PeerJ. 2023 Nov 29;11:e16384. doi: 10.7717/peerj.16384 (PMC10693237; doi:10.7717/peerj.16384)
Supplement: Supplemental Information 4 [file peerj-11-16384-s004.docx]

Table S2 Likert-scale questions of awarenss

| Question | Item | Score |
| --- | --- | --- |
| Do you know the definition of medication adherence? | Completely  Greatly  Mildly  Slightly  Not at all | 5  4  3  2  1 |
| What training have you received?(Excluding specialized courses such as Internal Medicine) |  |  |
| Academic literature. | Always  Frequently  Sometimes  Occasionally  Never | 5  4  3  2  1 |
| Online academic conferences. | Always  Frequently  Sometimes  Occasionally  Never | 5  4  3  2  1 |
| Onsite lectures and peer discussions. | Always  Frequently  Sometimes  Occasionally  Never | 5  4  3  2  1 |
| Participation of hypertension research. | Always  Frequently  Sometimes  Occasionally  Never | 5  4  3  2  1 |
| Implementation of patient education | Always  Frequently  Sometimes  Occasionally  Never | 5  4  3  2  1 |
| Refresher training. | Always  Frequently  Sometimes  Occasionally  Never | 5  4  3  2  1 |
| What tools do you know about assessing medication adherence？ |  |  |
| Scales such as MMAS-8. | Completely  Greatly  Mildly  Slightly  Not at all | 5  4  3  2  1 |
| Regulatory systems of medication | Completely  Greatly  Mildly  Slightly  Not at all | 5  4  3  2  1 |
| Detection of biochemical indicators. | Completely  Greatly  Mildly  Slightly  Not at all | 5  4  3  2  1 |

| Question | Item | Score |
| --- | --- | --- |
