# Supplementary material for: Hypertension doctors’ awareness and practice of medication adherence in hypertensive patients: a questionnaire-based survey
Source: PeerJ. 2023 Nov 29;11:e16384. doi: 10.7717/peerj.16384 (PMC10693237; doi:10.7717/peerj.16384)
Supplement: Supplemental Information 5 [file peerj-11-16384-s005.docx]

Table S3 Likert-scale questions of practice

| Question | Item | Score |
| --- | --- | --- |
| How often did you evaluate medication adherence? | Always  Frequently  Sometimes  Occasionally  Never | 5  4  3  2  1 |
| How often did you highlight the importance of medication adherence for hypertensive patients? | Always  Frequently  Sometimes  Occasionally  Never | 5  4  3  2  1 |
| What interventions to improve medication adherence have you used? |  |  |
| Apps that facilitate patients’ self-management of blood pressure | Always  Frequently  Sometimes  Occasionally  Never | 5  4  3  2  1 |
| Audio and video materials for missions. | Always  Frequently  Sometimes  Occasionally  Never | 5  4  3  2  1 |
| Answering the patients’ questions. | Always  Frequently  Sometimes  Occasionally  Never | 5  4  3  2  1 |
| Emphasis on the role of medication | Always  Frequently  Sometimes  Occasionally  Never | 5  4  3  2  1 |
| Regimens that minimize the number of doses. | Always  Frequently  Sometimes  Occasionally  Never | 5  4  3  2  1 |
| Requirements for regular outpatient follow-up. | Always  Frequently  Sometimes  Occasionally  Never | 5  4  3  2  1 |
| Requirements for escorts/families to observe the patient's medication. | Always  Frequently  Sometimes  Occasionally  Never | 5  4  3  2  1 |
| How often did you take individual differences into account when implementing interventions to improve medication adherence? | Always  Frequently  Sometimes  Occasionally  Never | 5  4  3  2  1 |
