# Supplementary material for: Hypertension doctors’ awareness and practice of medication adherence in hypertensive patients: a questionnaire-based survey
Source: PeerJ. 2023 Nov 29;11:e16384. doi: 10.7717/peerj.16384 (PMC10693237; doi:10.7717/peerj.16384)
Supplement: Supplemental Information 6 [file peerj-11-16384-s006.docx]

Table S4 Univariate analysis of awareness

| Demographic characteristic | Variables | Cognitive score  mean (SD) | Z/H* | *p* |
| --- | --- | --- | --- | --- |
|  |  |  |  |  |
| Gender | Male  female | 30.48 (9.23)  29.41 (8.53) | -0.856 | 0.392 |
| Age, years | ≤30  31-39  40-46  ≥47 | 23.95 (6.52)  29.76 (8.02)  31.35 (8.02)  34.81 (8.89) | 51.190 | **<0.001** |
| Work experience, years | ≤5  6-13  14-24  ≥25 | 24.24 (6.44)  29.74 (8.07)  31.32 (7.94)  34.38 (9.50) | 42.866 | **<0.001** |
| Education and training | Doctor's degree  Master's degree  Bachelor's degree and below | 38.27 (7.23)  28.99 (8.80)  28.24 (7.93) | 29.255 | **<0.001** |
| Hospital level | Provincial-level Grade III-A  City-level Grade III-A  Grade III-B  Grade II or below | 29.47 (9.01)  33.70 (9.09)  28.16 (7.72)  27.96 (7.89) | 13.17 | **0.004** |
| Professional ranks | Residents  Attending physicians  Chief physicians | 23.82 (6.49)  28.61 (7.33)  34.06 (8.63) | 57.219 | **<0.001** |
| History of hypertension | No  Yes | 29.22 (8.56)  33.90 (9.39) | 2.648 | **0.008** |
| Family history of hypertension | No  Yes | 28.66 (8.74)  30.75 (8.73) | 2.053 | **0.040** |
| the number of consulting for hypertension per week | ＞50  40-49  30-39  20-29  ＜20 | 45.65 (3.81)  38.41 (4.31)  32.68 (4.43)  29.38 (5.46)  22.12 (4.56) | 168.37 | **<0.001** |
| The number of antihypertensive prescriptions issued per week | ＞50  40-49  30-39  20-29  ＜20 | 48.17 (1.90)  39.81 (3.73)  35.60 (3.71)  31.49 (4.65)  23.34 (5.30) | 161.89 | **<0.001** |

*Z: Mann-Whitney U test; H: Kruskal-Wallis test
