# Supplementary material for: Hypertension doctors’ awareness and practice of medication adherence in hypertensive patients: a questionnaire-based survey
Source: PeerJ. 2023 Nov 29;11:e16384. doi: 10.7717/peerj.16384 (PMC10693237; doi:10.7717/peerj.16384)
Supplement: Supplemental Information 7 [file peerj-11-16384-s007.docx]

Table S5 Univariate analysis of practice

| Demographic characteristic | Variables | Practical score  mean (SD) | Z/H* | *p* |
| --- | --- | --- | --- | --- |
|  |  |  |  |  |
| Gender | Male  Female | 39.19 (7.08)  39.55 (7.18) | 0.469 | 0.639 |
| Age, years | ≤30  31-39  40-46  ≥47 | 34.35 (6.81)  40.16 (6.77)  40.61 (5.50)  43.11 (6.22) | 53.924 | **<0.001** |
| Work experience, years | ≤5  6-13  14-24  ≥25 | 33.85 (6.43)  40.28 (5.93)  41.24 (6.03)  42.76 (6.74) | 58.868 | **<0.001** |
| Education and training | Doctor's degree  Master's degree  Bachelor's degree and below | 43.43 (5.45)  37.91 (7.12)  39.53 (7.15) | 16.050 | **<0.001** |
| Hospital level | Provincial-level Grade III-A  City-level Grade III-A  Grade III-B  Grade II or below | 38.47 (7.34)  41.82 (5.64)  38.97 (6.83)  39.14 (7.83) | 7.095 | 0.069 |
| Professional ranks | Residents  Attending physicians  Chief physicians | 34.12 (6.58)  39.90 (6.56)  42.15 (6.12) | 52.901 | **<0.001** |
| History of hypertension | No  Yes | 38.78 (7.18)  44.03 (4.72) | 3.944 | **<0.001** |
| Family history of hypertension | No  Yes | 37.98 (7.39)  40.64 (6.69) | 2.668 | **0.008** |
| The number of consulting for hypertension per week | >50  40-49  30-39  20-29  <20 | 49.35 (1.22)  45.73 (3.14)  42.97 (2.57)  38.79 (3.83)  33.70 (6.39) | 152.70 | **<0.001** |
| The number of antihypertensive prescriptions issued per week | >50  40-49  30-39  20-29  <20 | 49.42 (1.44)  47.37 (2.26)  43.46 (3.38)  41.54 (3.22)  34.66 (6.25) | 143.16 | **<0.001** |

*Z: Mann-Whitney U test; H: Kruskal-Wallis test
