# Supplementary material for: Hypertension doctors’ awareness and practice of medication adherence in hypertensive patients: a questionnaire-based survey
Source: PeerJ. 2023 Nov 29;11:e16384. doi: 10.7717/peerj.16384 (PMC10693237; doi:10.7717/peerj.16384)
Supplement: Supplemental Information 8 [file peerj-11-16384-s008.pptx]

## Slide 1
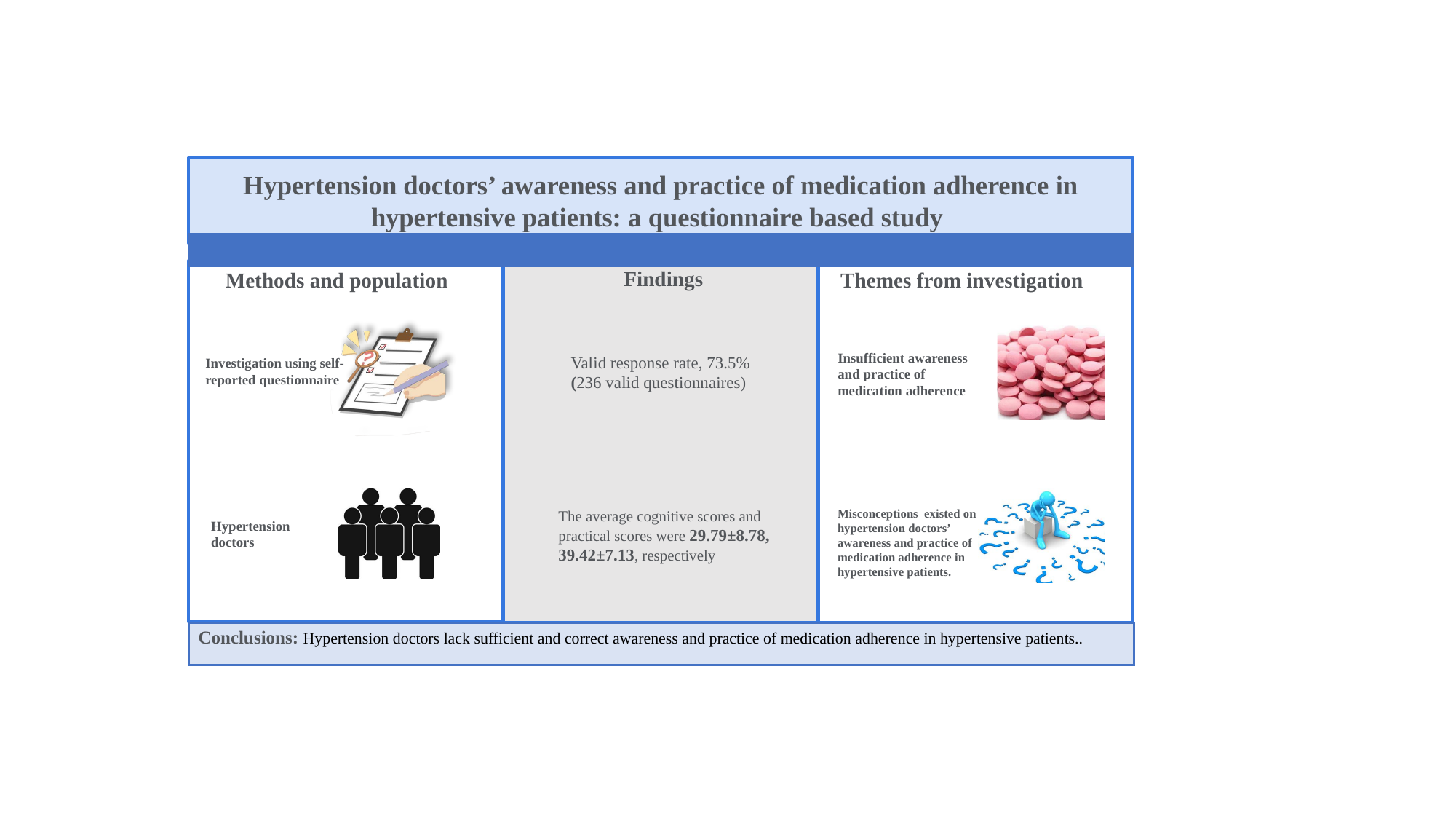

Hypertension doctors’ awareness and practice of medication adherence in hypertensive patients: a questionnaire based study
Findings
Methods and population
Themes from investigation
Insufficient awareness and practice of medication adherence
Valid response rate, 73.5% (236 valid questionnaires)
Investigation using self-reported questionnaire
Misconceptions existed on hypertension doctors’ awareness and practice of medication adherence in hypertensive patients.
The average cognitive scores and practical scores were 29.79±8.78, 39.42±7.13, respectively
Hypertension doctors
Conclusions: Hypertension doctors lack sufficient and correct awareness and practice of medication adherence in hypertensive patients..
